# Modern Sociology

**Published:** 1902-07-26

**Authors:** 


### Modern Sociology.

By a Correspondent.

### RURAL HOUSING.

While the housing of the poor in our large towns receives much of the attention of the public, that of the housing of the rural poor is almost forgotten. Yet those who live in the country know that the conditions of life in many villages are often as bad as that in city slums, although the purer air which the cottagers can enjoy outside their dwellings perhaps prevents the results from being so baneful.

This question of rural housing is certainly a difficult one. In many villages the supply of cottages does not equal the demand for them. A correspondent who has sent us much information on the subject—Miss Constance Cochrane, of St. Neot's—mentions that young people who are engaged to be married have to wait until some change in the village population enables them to get a home of any kind; and what accommodation there is in cottages is often insufficient. A family home should contain at least four apartments, a living room, and three sleeping rooms, one for the parents, one for sons, and one for daughters. It will be agreed that these are the simple demands of decency. And it must be admitted that these demands are but rarely complied with. A single bedroom is often all that is provided, where parents and children huddle together almost promiscuously. Can this condition of affairs be remedied? It is easy to say offhand that it ought to be, but one must look at the practical limitations. In a village where the population is stationary or diminishing, it is obvious that private enterprise will not incline to the building of new cottages. A man who invests money in house property does so where there is a fair prospect of a return for his money. And it is very doubtful if we can rightfully ask the local authorities to do what private enterprise shrinks from. The popular cry, so general now, that the State should do this often means only a desire to be charitable with other people's money. Local authorities cannot indulge in large capital expenditure in places where there is a prospect of the rateable income growing less. It is said that if better houses were provided, the rural population would not be so anxious to migrate to the towns. That remains to be proved. Certainly the housing of the poor in towns is not so good as in itself to induce rustics to go there if there were not also the hope of better wages and a livelier life. It may be that it would be a profitable venture for someone to build good new cottages in some of these villages, and certainly there often seems to be a lack of initiative in village residents which keeps them from trying a chance which lies at their door; but while the conditions of agricultural life remain as they are, it is to be feared that such building must be left rather to someone of philanthropic inclinations than to either private speculators or local authorities, of whom the first has not the will and the second not the right to risk losses. When the philanthropist has proved—as he might—that the venture pays, others will follow in his footsteps. But the first step must come from one who can afford, though he does not want to lose money For the question is not the provision of almshouses, but of decent cottages at a rent which the working classes in the country can pay, and which will give some return for the money invested.

But apart from this insufficiency of accommodation much might be done to improve the conditions of life in the country. Old cottages may exist without a proper water supply or sanitary conveniences, but why are new ones built which are in no way an improvement on the old? It is in this matter, rather than in that of undertaking new buildings, that we have a right to turn to the local authorities. Laws exist for the regulation of dwellings, but are they carried out? Sanitary inspectors are appointed, but are they always competent? We fear not. Where local wires can be pulled, the post may be given to some lazy or ignorant individual who is either past work or is incapable of earning his living in the open labour market. Miss Cochrane quotes one case where the sanitary inspector was a retired policeman quite ignorant of the work he was supposed to do. An official like this can nullify the best law that ever stood on a statute book. Again, the medical officer of health is often a medical practitioner with a widespread practice. He receives a small salary for his public health work, and cannot be expected to devote to it a large part of his time. Moreover, it is always possible that the property owners who are the greatest offenders against the law are among his private patients, and, without disparaging the honesty of the profession as a whole, it may be admitted that there are practitioners who will not risk offending, and perhaps losing, a patient for the sake of abstract right. Before asking for new powers from the State, and perhaps laying new burdens on the community, it is right to see that existing powers are carried out. A sanitary inspector without sanitary training should not be tolerated, and it is necessary, if he is to do his work fearlessly, that the medical officer of health should be independent of the local notables, and should be able to devote his whole time to public work. This matter of the independence of the sanitary officers is of the first importance. The Rev. Dr. Fry, of Berkhampstead, said at a conference on rural housing held in Sion College in last October: "As to medical officers of health, in all districts you will have to secure that they can only be dismissed by the Local Government Board. Medical officers of health have one after another told me that, because of their wives and families, they dare not do what they would otherwise willingly do if they were defended against attack."

Where the careful and strict administration of the law will meet the case, the question of housing is not insoluble. It must be admitted that when new building has to be faced, and there is no member of the village community willing to do it, the thing is more serious. At the conference we have mentioned, Mr. Bolton King, of Gaydon, near Warwick, declared that "if cottages at all suitable are built, there must be a loss." This loss he reckons at "something like 1½ per cent." on every cottage. Miss Escombe, a parish councillor from Penshurst, was able to show that in her neighbourhood it had been possible for the District Council to build cottages by means of a loan at 3½ per cent. for 40 years from the Local Government Board, which added to the rates only 1d. in the pound for a single year.

There is another difficulty on which we have not touched. As the Archbishop of Canterbury said at a Church Congress, "If we could get the poor to co-operate with us in our endeavours to raise them, we could do ten times more.' This is a real obstruction. Many who could afford better accommodation are content to be crowded in small and insanitary dwellings. Until we raise the standard of living among the people themselves, it is to be feared that the housing of the poor and the working classes both in town and country will always present great difficulties. Meanwhile, it might be helpful if the Mansion House Committee, the most practical committee, perhaps, which has ever undertaken the matter, could extend its action into the country districts, beginning with the home counties, from which the good example might spread to districts more remote.

